# Measurement and analysis of change in research scholars’ knowledge and attitudes toward statistics after PhD coursework

**DOI:** 10.1186/s12909-024-05487-y

**Published:** 2024-05-08

**Authors:** Mariyamma Philip

**Affiliations:** Department of Biostatistics, Dr. M.V. Govindaswamy Centre, National Institute of Mental Health and Neurosciences (NIMHANS), Bangalore, 560 029 India

**Keywords:** Knowledge of statistics, Attitude towards statistics, PhD coursework, Research scholars

## Abstract

**Background:**

Knowledge of statistics is highly important for research scholars, as they are expected to submit a thesis based on original research as part of a PhD program. As statistics play a major role in the analysis and interpretation of scientific data, intensive training at the beginning of a PhD programme is essential. PhD coursework is mandatory in universities and higher education institutes in India. This study aimed to compare the scores of knowledge in statistics and attitudes towards statistics among the research scholars of an institute of medical higher education in South India at different time points of their PhD (i.e., before, soon after and 2–3 years after the coursework) to determine whether intensive training programs such as PhD coursework can change their knowledge or attitudes toward statistics.

**Methods:**

One hundred and thirty research scholars who had completed PhD coursework in the last three years were invited by e-mail to be part of the study. Knowledge and attitudes toward statistics before and soon after the coursework were already assessed as part of the coursework module. Knowledge and attitudes towards statistics 2–3 years after the coursework were assessed using Google forms. Participation was voluntary, and informed consent was also sought.

**Results:**

Knowledge and attitude scores improved significantly subsequent to the coursework (i.e., soon after, percentage of change: 77%, 43% respectively). However, there was significant reduction in knowledge and attitude scores 2–3 years after coursework compared to the scores soon after coursework; knowledge and attitude scores have decreased by 10%, 37% respectively.

**Conclusion:**

The study concluded that the coursework program was beneficial for improving research scholars’ knowledge and attitudes toward statistics. A refresher program 2–3 years after the coursework would greatly benefit the research scholars. Statistics educators must be empathetic to understanding scholars’ anxiety and attitudes toward statistics and its influence on learning outcomes.

## Background

A PhD degree is a research degree, and research scholars submit a thesis based on original research in their chosen field. Doctor of Philosophy (PhD) degrees are awarded in a wide range of academic disciplines, and the PhD students are usually referred as research scholars. A comprehensive understanding of statistics allows research scholars to add rigour to their research. This approach helps them evaluate the current practices and draw informed conclusions from studies that were undertaken to generate their own hypotheses and to design, analyse and interpret complex clinical decisions. Therefore, intensive training at the beginning of the PhD journey is essential, as intensive training in research methodology and statistics in the early stages of research helps scholars design and plan their studies efficiently.

The University Grants Commission of India has taken various initiatives to introduce academic reforms to higher education institutions in India and mandated in 2009 that coursework be treated as a prerequisite for PhD preparation and that a minimum of four credits be assigned to one or more courses on research methodology, which could cover areas such as quantitative methods, computer applications, and research ethics. UGC also clearly states that all candidates admitted to PhD programmes shall be required to complete the prescribed coursework during the initial two semesters [1]. National Institute of Mental Health and Neurosciences (NIMHANS) at Bangalore, a tertiary care hospital and medical higher education institute in South India, that trains students in higher education in clinical fields, also introduced coursework in the PhD program for research scholars from various backgrounds, such as basic, behavioral and neurosciences, as per the UGC mandate. Research scholars undertake coursework programs soon after admission, which consist of several modules that include research methodology and statistical software training, among others.

Most scholars approach a course in statistics with the prejudice that statistics is uninteresting, demanding, complex or involve much mathematics and, most importantly, it is not relevant to their career goals. They approach statistics with considerable apprehension and negative attitudes, probably because of their inability to grasp the relevance of the application of the methods in their fields of study. This could be resolved by providing sufficient and relevant examples of the application of statistical techniques from various fields of medical research and by providing hands-on experience to learn how these techniques are applied and interpreted on real data. Hence, research methodology and statistical methods and the application of statistical methods using software have been given much importance and are taught as two modules, named Research Methodology and Statistics and Statistical Software Training, at this institute of medical higher education that trains research scholars in fields as diverse as basic, behavioural and neurosciences. Approximately 50% of the coursework curriculum focused on these two modules. Research scholars were thus given an opportunity to understand the theoretical aspects of the research methodology and statistical methods. They were also given hands-on training on statistical software to analyse the data using these methods and to interpret the findings. The coursework program was designed in this specific manner, as this intensive training would enable the research scholars to design their research studies more effectively and analyse their data in a better manner.

It is important to study attitudes toward statistics because attitudes are known to impact the learning process. Also, most importantly, these scholars are expected to utilize the skills in statistics and research methods to design research projects or guide postgraduate students and research scholars in the near future. Several authors have assessed attitudes toward statistics among various students and examined how attitudes affect academic achievement, how attitudes are correlated with knowledge in statistics and how attitudes change after a training program. There are studies on attitudes toward statistics among graduate [2–4] and postgraduate [5] medical students, politics, sociology, (6–7) psychology [8–10], social work [11], and management students [12]. However, there is a dearth of related literature on research scholars, and there are only two studies on the attitudes of research scholars. In their study of doctoral students in education-related fields, Cook & Catanzaro (2022) investigated the factors that contribute to statistics anxiety and attitudes toward statistics and how anxiety, attitudes and plans for future research use are connected among doctoral students [13]. Another study by Sohrabi et al. (2018) on research scholars assessed the change in knowledge and attitude towards teaching and educational design of basic science PhD students at a Medical University after a two-day workshop on empowerment and familiarity with the teaching and learning principles [14]. There were no studies that assessed changes in the attitudes or knowledge of research scholars across the PhD training period or after intensive training programmes such as PhD coursework. Even though PhD coursework has been established in institutes of higher education in India for more than a decade, there are no published research on the effectiveness of coursework from Indian universities or institutes of higher education.

This study aimed to determine the effectiveness of PhD coursework and whether intensive training programs such as PhD coursework can influence the knowledge and attitudes toward statistics of research scholars. Additionally, it would be interesting to know if the acquired knowledge could be retained longer, especially 2–3 years after the coursework, the crucial time of PhD data analysis. Hence, this study compares the scores of knowledge in statistics and attitude toward statistics of the research scholars at different time points of their PhD training, i.e., before, soon after and 2–3 years after the coursework.

## Methods

### Participants

This is an observational study of single group with repeated assessments. The institute offers a three-month coursework program consisting of seven modules, the first module is ethics; the fifth is research methodology and statistics; and the last is neurosciences. The study was conducted in January 2020. All research scholars of the institute who had completed PhD coursework in the last three years were considered for this study (*n* = 130). Knowledge and attitudes toward statistics before and soon after the coursework module were assessed as part of the coursework program. They were collected on the first and last day of the program respectively. The author who was also the coordinator of the research methodology and statistics module of the coursework have obtained the necessary permission to use the data for this study. The scholars invited to be part of the study by e-mail. Knowledge and attitude towards statistics 2–3 years after the coursework were assessed online using Google forms. They were also administered a semi structured questionnaire to elicit details about the usefulness of coursework. Participation was voluntary, and consent was also sought online. The confidentiality of the data was assured. Data were not collected from research scholars of Biostatistics or from research scholars who had more than a decade of experience or who had been working in the institute as faculty, assuming that their scores could be higher and could bias the findings. This non funded study was reviewed and approved by the Institute Ethics Committee.

### Instruments

Knowledge in Statistics was assessed by a questionnaire prepared by the author and was used as part of the coursework evaluation. The survey included 25 questions that assessed the knowledge of statistics on areas such as descriptive statistics, sampling methods, study design, parametric and nonparametric tests and multivariate analyses. Right answers were assigned a score of 1, and wrong answers were assigned a score of 0. Total scores ranged from 0 to 25. Statistics attitudes were assessed by the Survey of Attitudes toward Statistics (SATS) scale. The SATS is a 36-item scale that measures 6 domains of attitudes towards statistics. The possible range of scores for each item is between 1 and 7. The total score was calculated by dividing the summed score by the number of items. Higher scores indicate more positive attitudes. The SAT-36 is a copyrighted scale, and researchers are allowed to use it only with prior permission. (15–16) The author obtained permission for use in the coursework evaluation and this study. A semi structured questionnaire was also used to elicit details about the usefulness of coursework.

### Statistical analysis

Descriptive statistics such as mean, standard deviation, number and percentages were used to describe the socio-demographic data. General Linear Model Repeated Measures of Analysis of variance was used to compare knowledge and attitude scores across assessments. Categorical data from the semi structured questionnaire are presented as percentages. All the statistical tests were two-tailed, and a p value < 0.05 was set a priori as the threshold for statistical significance. IBM SPSS (28.0) was used to analyse the data.

## Results

One hundred and thirty research scholars who had completed coursework (CW) in the last 2–3 years were considered for the study. These scholars were sent Google forms to assess their knowledge and attitudes 2–3 years after coursework. 81 scholars responded (62%), and 4 scholars did not consent to participate in the study. The data of 77 scholars were merged with the data obtained during the coursework program (before and soon after CW). Socio-demographic characteristics of the scholars are presented in Table 1.

**Table 1:** Socio-demographic characteristics of the scholars

Variables		Mean ± SD / Median (Range)
Age in years		28.7 ± 3.01 (23–36)
Years of experience		3 (0.5–9)
		Number (%)
Gender	Males	27 (35%)
	Females	50 (65%)
Division	Basic sciences	22 (29%)
	Behavioural sciences	43 (55%)
	Neuro sciences	12 (16%)

The age of the respondents ranged from 23 to 36 years, with an average of 28.7 years (3.01), and the majority of the respondents were females (65%). Years of experience (i.e., after masters) before joining a PhD programme ranged from 0.5 to 9 years, and half of them had less than three years of experience before joining the PhD programme (median-3). More than half of those who responded were research scholars from the behavioural sciences (55%), while approximately 30% were from the basic sciences (29%).

**Fig. 1:**
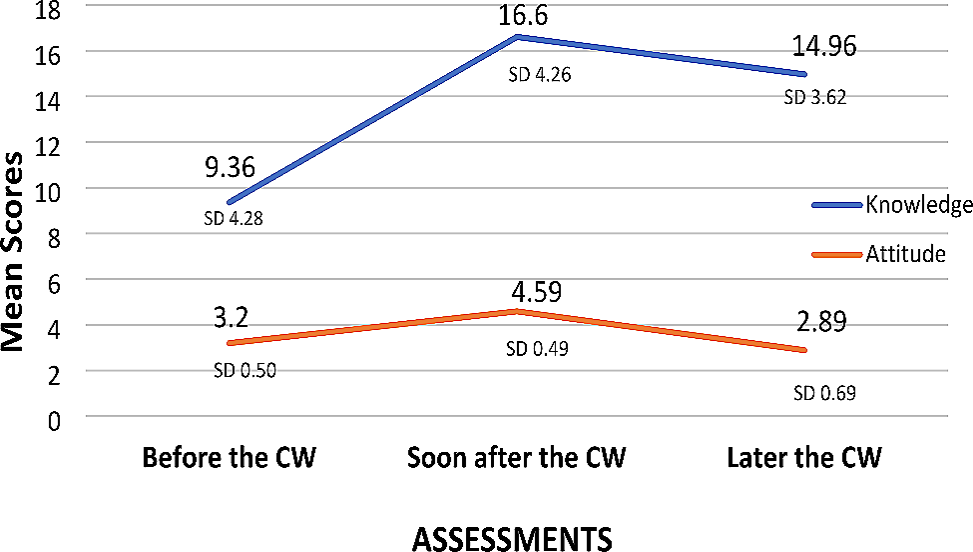
Comparison of knowledge and attitude scores across the assessments. Later the CW – 2–3 years after the coursework

General Linear Model Repeated Measures of Analysis of variance was used to compare the knowledge and attitude scores of scholars before, soon after and 2–3 after the coursework (will now be referred as “later the CW”), and the results are presented below (Table 2; Fig. 1).

**Table 2:** Comparison of knowledge and attitude scores across the assessments

Variables	ASSESSMENTS			F- value (*p*-value)	Effect size*
	Before the CW	Soon after the CW	Later the CW^@^		
	Mean ± SD				
Knowledge score	9.36 ± 4.28	16.60^#^ ± 4.26	14.96^$^ ± 3.62	107.34 (0.001)	0.585
Attitude scores	3.20 ± 0.50	4.59^#^ ± 0.49	2.89^$^ ± 0.69	492.71 (0.001)	0.866

@ Later the CW – 2–3 years after the coursework

# Scores “Soon after the CW” were significantly greater than scores “Before the CW”

$ Scores “Later the CW” were significantly lower than scores obtained both at “Before CW” and “Soon after CW”

* Partial Eta square (η_p_²) values

The scores for knowledge and attitude differed significantly across time. Scores of knowledge and attitude increased soon after the coursework; the percentage of change was 77% and 43% respectively. However, significant reductions in knowledge and attitude scores were observed 2–3 years after the coursework compared to scores soon after the coursework. The reduction was higher for attitude scores; knowledge and attitude scores have decreased by 10% and 37% respectively. The change in scores across assessments is evident from the graph, and clearly the effect size is higher for attitude than knowledge.

The scores of knowledge or attitude before the coursework did not significantly differ with respect to gender or age or were not correlated with years of experience. Hence, they were not considered as covariates in the above analysis.

**Fig. 2:**
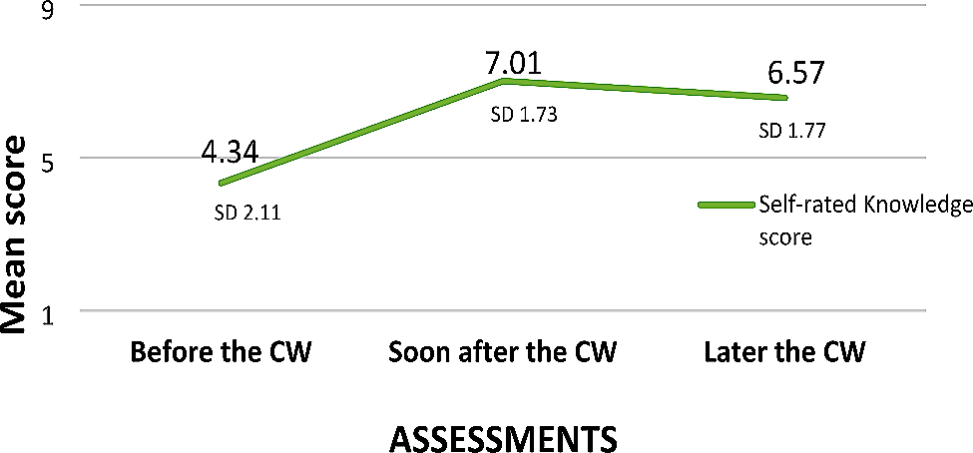
Self-rated knowledge scores of research scholars over time. Later the CW – 2–3 years after the coursework

A semi structured questionnaire with open ended questions was also administered to elicit in-depth information about the usefulness of the coursework programme, in which they were also asked to self- rate their knowledge. The data were mostly categorical or narratives. Research scholars’ self-rated knowledge scores (on a scale of 0–10) also showed similar changes; knowledge improved significantly and was retained even after the training (Fig. 2).

**Fig. 3:**
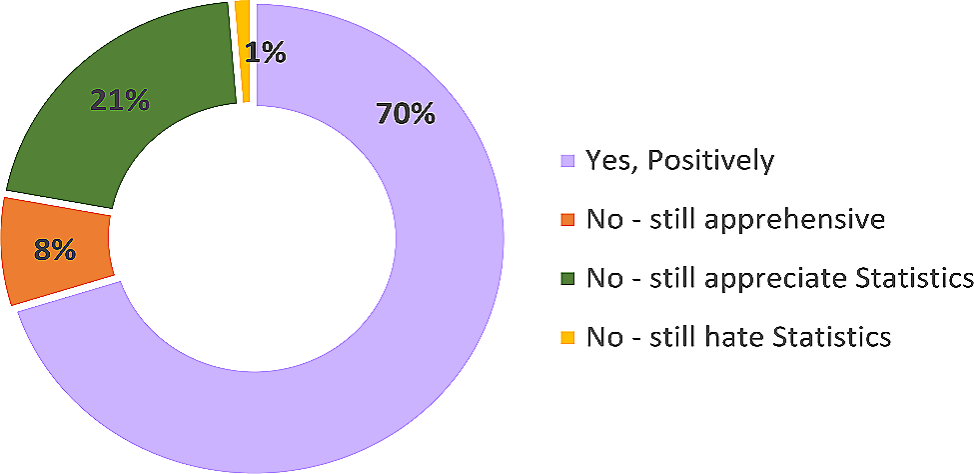
How has coursework changed your attitude toward statistics?

The response to the question “*How has coursework changed your attitude* toward statistics?”, is presented in Fig. 3. The responses were Yes, positively, Yes - Negatively, No change – still apprehensive, No change – still appreciate, No change – still hate statistics. The majority of the scholars (70%) reported a positive change in their attitude toward statistics. Moreover, none of the scholars reported negative changes. Approximately 9% of the scholars reported that they were still apprehensive about statistics or hate statistics after the coursework.

Those scholars who reported that they were apprehensive about statistics or hate statistics noted the complexity of the subject, lack of clarity, improper instructions and fear of mathematics as major reasons for their attitude. Some responses are listed below.

*“The statistical concepts were not taught in an understandable manner from the UG level”*,

*“I am weak in mathematical concepts. The equations and formulae in statistics scare me”.*

*“Lack of knowledge about the importance of statistics and fear of mathematical equations”.*

*“The preconceived notion that Statistics is difficult to learn”*.

*“In most of the places, it is not taught properly and conceptual clarity is not focused on, and because of this an avoidance builds up, which might be a reason for the negative attitude”.*

Majority of the scholars (92%) felt that coursework has helped them in their PhD, and they were happy to recommend it for other research scholars (97%). The responses of the scholars to the question “*How was coursework helpful in your PhD journey*?”, are listed below.

*“Course work gave a fair idea on various things related to research as well as statistics”*.

*“Creating the best design while planning methodology, which is learnt form course work, will increase efficiency in completing the thesis, thereby making it faster”.*

*“Course work give better idea of how to proceed in many areas like literature search, referencing, choosing statistical methods, and learning about research procedures”.*

*“Course work gave a good idea of research methodology, biostatistics and ethics. This would help in writing a better protocol and a better thesis”.*

*“It helps us to plan our research well and to formulate, collect and plan for analysis”.*

*“It makes people to plan their statistical analysis well in advance”*.

## Discussion

This study evaluated the effectiveness of the existing coursework programme in an institution of higher medical education, and investigated whether the coursework programme benefits research scholars by improving their knowledge of statistics and attitudes towards statistics. The study concluded that the coursework program was beneficial for improving scholars’ knowledge about statistics and attitudes toward statistics.

Unlike other studies that have assessed attitudes toward statistics, the study participants in this study were research scholars. Research scholars need extensive training in statistics, as they need to apply statistical tests and use statistical reasoning in their research thesis, and in their profession to design research projects or their future student dissertations. Notably, no studies have assessed the attitudes or knowledge of research scholars in statistics either across the PhD training period or after intensive statistics training programs. However, the findings of this study are consistent with the findings of a study that compared the knowledge and attitudes toward teaching and education design of PhD students after a two-day educational course and instructional design workshop [14]. 

Statistics educators need not only impart knowledge but they should also motivate the learners to appreciate the role of statistics and to continue to learn the quantitative skills that is needed in their professional lives. Therefore, the role of learners’ attitudes toward statistics requires special attention. Since PhD coursework is possibly a major contributor to creating a statistically literate research community, scholars’ attitudes toward statistics need to be considered important and given special attention. Passionate and engaging statistics educators who have adequate experience in illustrating relatable examples could help scholars feel less anxious and build competence and better attitudes toward statistics. Statistics educators should be aware of scholars’ anxiety, fears and attitudes toward statistics and about its influence on learning outcomes and further interest in the subject.

### Strengths and limitations

Analysis of changes in knowledge and attitudes scores across various time points of PhD training is the major strength of the study. Additionally, this study evaluates the effectiveness of intensive statistical courses for research scholars in terms of changes in knowledge and attitudes. This study has its own limitations: the data were collected through online platforms, and the nonresponse rate was about 38%. Ability in mathematics or prior learning experience in statistics, interest in the subject, statistics anxiety or performance in coursework were not assessed; hence, their influence could not be studied. The reliability and validity of the knowledge questionnaire have not been established at the time of this study. However, author who had prepared the questionnaire had ensured questions from different areas of statistics that were covered during the coursework, it has also been used as part of the coursework evaluation. Despite these limitations, this study highlights the changes in attitudes and knowledge following an intensive training program. Future research could investigate the roles of age, sex, mathematical ability, achievement or performance outcomes and statistics anxiety.

## Conclusion

The study concluded that a rigorous and intensive training program such as PhD coursework was beneficial for improving knowledge about statistics and attitudes toward statistics. However, the significant reduction in attitude and knowledge scores after 2–3 years of coursework indicates that a refresher program might be helpful for research scholars as they approach the analysis stage of their thesis. Statistics educators must develop innovative methods to teach research scholars from nonstatistical backgrounds. They also must be empathetic to understanding scholars’ anxiety, fears and attitudes toward statistics and to understand its influence on learning outcomes and further interest in the subject.

## Data Availability

The data that support the findings of this study are available from the corresponding author upon request.
